# The Family History of Developmental Stuttering: A Scoping Review

**DOI:** 10.1111/1460-6984.70323

**Published:** 2026-08-31

**Authors:** Yuto Sato, Mizuki Aoki, Shoko Miyamoto, Daichi Iimura

**Affiliations:** ^1^ Graduate School of Comprehensive Human Sciences University of Tsukuba Tsukuba‐city Ibaraki Japan; ^2^ Institute of Human Sciences University of Tsukuba Tsukuba‐city Ibaraki Japan

**Keywords:** epidemiology, family history, scoping review, stuttering

## Abstract

**Background:**

A positive family history is a significant risk factor for developmental stuttering. However, the reported rates of positive family histories are inconsistent across studies, making it difficult to synthesise the findings.

**Aim:**

This scoping review aimed to systematically map the literature on family histories of stuttering to clarify the reported stuttering proportions and identify methodological trends and gaps.

**Method & Procedures:**

We conducted a literature search of PubMed, Web of Science, and PsycINFO on 4 February 2025. Two authors independently screened the records in two stages: first by title and abstract, and then by full text.

**Outcomes & Results:**

We reviewed 19 studies that reported family histories of participants with developmental stuttering. Basic study characteristics such as sample size and country were extracted, and descriptive statistics, including family history rates, were calculated for quantitative data. A key finding was the wide variability in the reported proportion of individuals with a positive family history, ranging from 20.0% to 90.9%. This variability was strongly associated with inconsistencies in methodology, including the scope of relatives considered, the definition of stuttering within the family, and data collection methods.

**Conclusions & Implications:**

This review found that methodological variability appeared to be the primary factor contributing to the wide variation in reported rates in the research on family histories of stuttering. The findings suggest the need for rigorous research approaches to obtain a more comprehensive understanding of this phenomenon.

**WHAT THIS PAPER ADDS:**

*What is already known on this subject*

- A positive family history is widely recognized as a major risk factor for developmental stuttering, and genetic evidence suggests a strong heritable component. However, reported proportions of individuals with a family history of stuttering vary substantially across studies, making it difficult to synthesize the evidence and interpret findings consistently.

*What this study adds to the existing knowledge*

- This scoping review systematically mapped 19 studies and revealed that reported rates of positive family history range widely from 20.0% to 90.9%. The review identifies key sources of methodological variability as primary contributors to this inconsistency. These findings highlight the need for greater clarification when defining and assessing family history in stuttering research.

*What are the potential clinical implications of this study?*

- Clinicians and researchers may benefit from using clearer criteria and consistent reporting frameworks, which could support more accurate risk evaluation and better integration of genetic, epidemiological, and clinical evidence.

## 1 Introduction

Developmental stuttering is a speech fluency disorder. It has an incidence of over 5% of the population between the ages of two and five years, with a prevalence of over 1% in school‐aged children and older individuals worldwide (Abou Ella et al. 2015; Boyce et al. 2022; Cavenagh et al. 2015; Gürbüz Özgür and Özgür 2019; Kefalianos et al. 2017; Mohammadi and Bakhtiary 2024; Mohammadi et al. 2016; Nandhini Devi et al. 2018; Reilly et al. 2009; Reilly et al. 2013; Sakai et al. 2024; Singer et al. 2022; Viswanath et al. 2004). Approximately 60–80% of children naturally recover from stuttering within three years of onset (Cavenagh et al. 2015; Darmody et al. 2022; Gürbüz Özgür and Özgür 2019; Mohammadi and Bakhtiary 2024; Mohammadi et al. 2016; Nandhini Devi et al. 2018; Singer et al. 2022). Because stuttering disrupts the smooth flow of speech and can make verbal communication challenging, early identification and intervention are important. In this context, information about an individual's family history may provide clinicians with an illuminating background for considering the risk and possible developmental course of stuttering, including its persistence and likelihood of recovery. Such information may also serve as a useful reference when clinicians assess individual risks and provide counselling to families (Yairi and Ambrose 2013). Among the various risk factors, a positive family history of stuttering is an important predictor of persistence, as stuttering is a highly heritable disorder (Boyce et al. 2022; Costa et al. 2022; Donaher and Richels 2012; Gürbüz Özgür and Özgür 2019; Kefalianos et al. 2017; Mohammadi and Bakhtiary 2024; Mohammadi et al. 2016; Sakai et al. 2024; Singer et al. 2022; Viswanath et al. 2004; Walsh et al. 2021). Understanding of its aetiology has advanced, with genetic factors found to account for over 70% of cases and with a positive family history often observed (Boyce et al. 2022; Choi et al. 2018; Darmody et al. 2022; Mohammadi and Bakhtiary 2024). Furthermore, anomalies in the white matter of the brain and resulting functional connectivity deficits between language‐related brain regions have been reported (Darmody et al. 2022; Donaher and Richels 2012; Gürbüz Özgür and Özgür 2019). The symptoms of stuttering are not limited to speech impairment but also have serious psychosocial consequences. People who stutter may experience teasing and bullying from peers, which can lead to feelings of intense shame as well as high comorbidity with social anxiety disorders and depression (Boyce et al. 2022; Darmody et al. 2022; Gürbüz Özgür and Özgür 2019; Mohammadi and Bakhtiary 2024; Reilly et al. 2009; Singer et al. 2022). Such outcomes can lead to speech‐avoidance behaviours, which may, in turn, affect overall productivity and employability. For example, a large‐scale study found that people who stutter in the United States earn significantly less than their fluent counterparts, which could contribute to a significant decrease in their quality of life (Gerlach et al. 2018). Research on the aetiology of stuttering has advanced dramatically in recent years, particularly in the field of genetics. Landmark studies have identified several candidate genes associated with stuttering (e.g. GNPTAB, GNPTG, NAGPA, and AP4E1), which has contributed significantly to improved understanding of its biological basis (Boyce et al. 2022; Choi et al. 2018; Darmody et al. 2022; Nandhini Devi et al. 2018). Efforts to elucidate the complex pathophysiology of stuttering and develop more effective interventions are ongoing.

The role of family history in the onset of stuttering has long been widely investigated (Kang 2015). Methodological approaches for defining and ascertaining family history vary considerably across studies (Choi et al. 2018). For example, the scope of relatives included ranges from only first‐degree relatives, such as parents and siblings (Kloth et al. 1998), to second‐degree relatives (e.g. grandparents and uncles), or third‐degree relatives (e.g. cousins) (MacFarlane et al. 1991; Poulos and Webster 1991). Furthermore, the criteria defining a positive family history are often ambiguous, as studies do not always clearly specify whether they include relatives who have ever stuttered (lifetime incidence) or only those currently stuttering (prevalence; Wepman 1939). Data collection methods are also diverse and include parent‐report questionnaires (Kloth et al. 1998), direct interviews (MacFarlane et al. 1991; Poulos and Webster 1991; Wepman 1939), and telephone reports (MacFarlane et al. 1991; Poulos and Webster 1991). These inconsistencies contribute to the wide variation in the reported rates of a positive family history of stuttering across these studies. Specifically, the lack of consistency in the definitions of family history and data collection methods across the literature makes integration of findings difficult (Kefalianos et al. 2017). Furthermore, these studies are geographically biased toward specific regions (e.g. the United States and Australia), which poses a significant challenge to achieving a comprehensive understanding of the epidemiology and aetiology of the disorder (Sakai et al. 2024). As linguistic and cultural factors can influence the manifestations and psychosocial impacts of stuttering, findings from a limited set of populations, primarily from English‐speaking Western countries, may not be generalisable. Moreover, this lack of diversity hinders a deeper understanding of potential gene‐environment interactions across various ancestral and sociocultural backgrounds. Given this situation, literature reviews that comprehensively organise the existing knowledge and identify research gaps are increasingly needed.

Therefore, this study aimed to conduct a scoping review to systematise the literature on developmental stuttering and family history, which entailed: consolidating existing knowledge regarding the proportion of individuals with a positive family history; identifying the factors related to such a family history; ascertaining trends in research design and assessment methods in family history studies; and determining existing research gaps and future research questions.

## 2 Materials and Methods

### 2.1 Information Sources and Search Strategy

This scoping review adhered to the Preferred Reporting Items for Systematic Reviews and Meta‐Analyses extension for Scoping Reviews (PRISMA‐ScR; Tricco et al. 2018), an extension of the PRISMA Statement (Moher et al. 2009; Page et al. 2021). To minimise selection bias, a comprehensive literature search was conducted, followed by systematic application of predefined inclusion and exclusion criteria. A literature search was conducted using three electronic databases: PubMed, Web of Science, and PsycINFO. The literature search was conducted using the following comprehensive search string: (stutter* OR stammer*) AND (“family history” OR “histories” OR epidemiolog* OR genetic* OR hereditary OR “genetic predisposition” OR “genetic mutation” OR “genetic susceptibility” OR “genetic correlation” OR “familial transmission” OR “genetic linkage” OR “epigenetic” OR “mutation analysis”). The same search string was used across all three databases, and only free‐text terms were used; no database‐specific thesaurus terms were included. The search was conducted on 4 February, 2025. All identified citations were imported into Rayyan systematic review software (Rayyan Systems Inc., Cambridge, MA, USA), and duplicates were automatically detected and manually removed.

### 2.2 Inclusion and Exclusion Criteria

To be eligible for inclusion, studies had to meet the following criteria: (a) report on the family history of the participants, including any information on twins or siblings; and (b) include more than one participant (i.e. not be a single‐case report). Studies were excluded if they met one or more of the following criteria: (a) were not original research, such as commentaries, reviews, dissertations, or conference abstracts; (b) did not include keywords related to speech disfluency (e.g. ‘stutter*’, ‘cluttering’, ‘disfluency’) in the title or abstract; (c) focused primarily on a condition other than developmental stuttering; (d) did not report on the family history of the participants; (e) were single‐case studies or did not report group‐level analysis; (f) were written in a language other than English; or (g) were duplicate publications.

### 2.3 Study Selection

**FIGURE 1:**
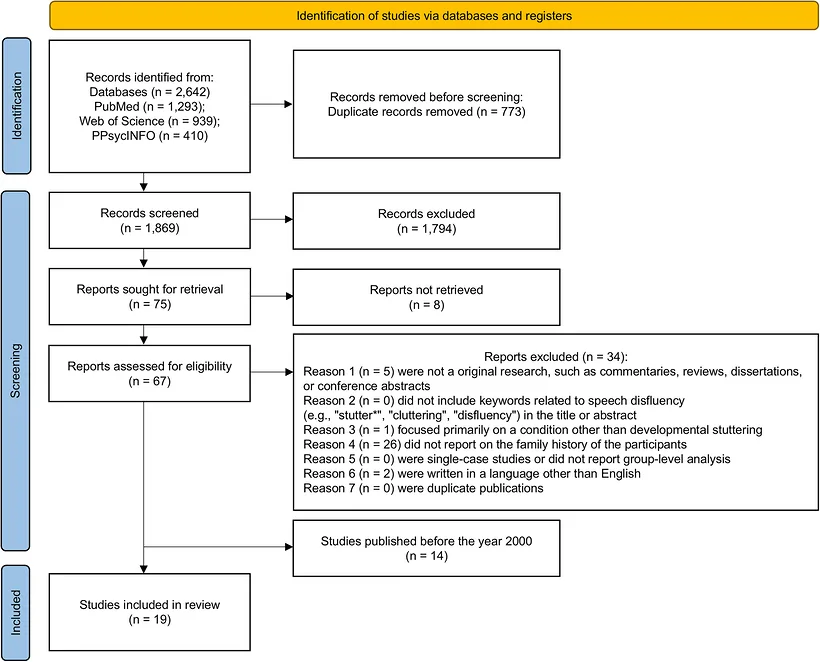
Flowchart of the PRISMA review process.

Following the PRISMA 2020 guidelines (Page et al. 2021), all identified records underwent a two‐stage selection process (Figure 1): first, a screening of titles and abstracts (Stage 1), and second, a full‐text review of the remaining articles to determine final eligibility (Stage 2). In Stage 1, two authors (M.A. and Y.S.) independently screened the titles and abstracts of all identified records against the eligibility criteria. In Stage 2, the same two authors independently assessed the full texts of the remaining articles to determine the final inclusion. Any disagreements between them at either stage were resolved through discussion. If a consensus was not reached, the third author (D.I.) was consulted for the final decision.

### 2.4 Data Items and Charting Process

The data items to be extracted were first determined through discussions among all three authors, and a corresponding data‐charting form was created. The extracted data were grouped into four primary categories: (a) general and study information; (b) participant characteristics; (c) stuttering onset and definition; and (d) family history data. The charted information consisted of binary, categorical, and numerical data.

All data charting and coding was conducted by one the authors (Y.S.). To ensure accuracy and consistency, the entire charting and coding process was reviewed and verified by a second author (M.A.). Any discrepancies identified during the review were resolved through discussions between these two authors.

To ensure methodological rigour, several specific coding rules were established a priori. First, to ensure consistency in coding the family history, a positive family history of stuttering was defined as a participant reporting at least one relative who stuttered. Second, the determination of stuttering presence was based solely on its prevalence at the time of the proband (primary participant) enrolment in the study. By contrast, as the criteria for their relatives were expected to be inconsistent across studies, we did not restrict the criteria for stuttering presence based on the current status. Some studies defined a positive family history based on lifetime incidence (including those who recovered; Buck et al. 2002; Cavenagh et al. 2015; Choi et al. 2018; Mohammadi and Bakhtiary 2024; Sakai et al. 2024), while others only considered relatives currently stuttering (Singer et al. 2022; Viswanath et al. 2004).

### 2.5 Data Charting and Summarising

The extracted data were collated and summarised in a narrative format to map the existing literature and identify key findings and research gaps. The key characteristics of the included studies were presented in a tabular format, distinguished by the study population (i.e. studies with controls vs. those with persons who stutter (PWS) only). The geographic distribution of the research was illustrated using a global map. Frequencies and percentages were used to summarise categorical data such as study design and country of origin. Descriptive statistics of the findings across the studies were reported for key numerical data, such as the rate of a positive family history. Specifically, the mean values were calculated as unweighted averages across the included studies. This approach was chosen to ensure treatment of each study as an equal unit of analysis and that the descriptive summary would reflect the breadth of the literature without being disproportionately influenced by a small number of studies with larger sample sizes.

## 3 Results

### 3.1 Study Selection

The literature search and selection process is summarised in the PRISMA flow diagram (Figure 1). The initial search across three electronic databases (PubMed, Web of Science, and PsycInfo) yielded 2642 citations. After removing 773 duplicates, 1869 articles remained for the initial screening of titles and abstracts. In the first stage, 1794 articles were excluded, leaving 75 articles for full‐text assessment. Of these, eight were excluded because their full text was not retrievable. The remaining 67 articles underwent a full‐text review, resulting in the exclusion of 34 articles. The interrater reliability between the two authors, measured using Cohen's kappa, was 0.441 for the first screening (moderate agreement) and 0.549 for the second screening (moderate agreement), according to the criteria proposed by Landis and Koch (1977). Discrepancies were resolved through discussions with the third author. The most common reason for exclusion at this stage was the absence of information on the participants’ family history (*n* = 26). Finally, 33 studies met all eligibility criteria. To identify contemporary research trends, this exploratory review focuses on studies published from 2000 onwards. After applying the publication‐year criterion, 19 studies were included in the final analysis. Among these 19 studies, three were published in the 2000s, nine in the 2010s, and seven in the early 2020s. Among the excluded pre‐2000 studies (*n* = 14), approximately 20% were twin studies, whereas none of the studies included in the final post‐2000 sample used this design. Given their different aims and sampling structures, the inclusion of twin studies may have complicated comparisons of reported family history rates across studies.

### 3.2 Characteristics of the Included Studies

**FIGURE 2:**
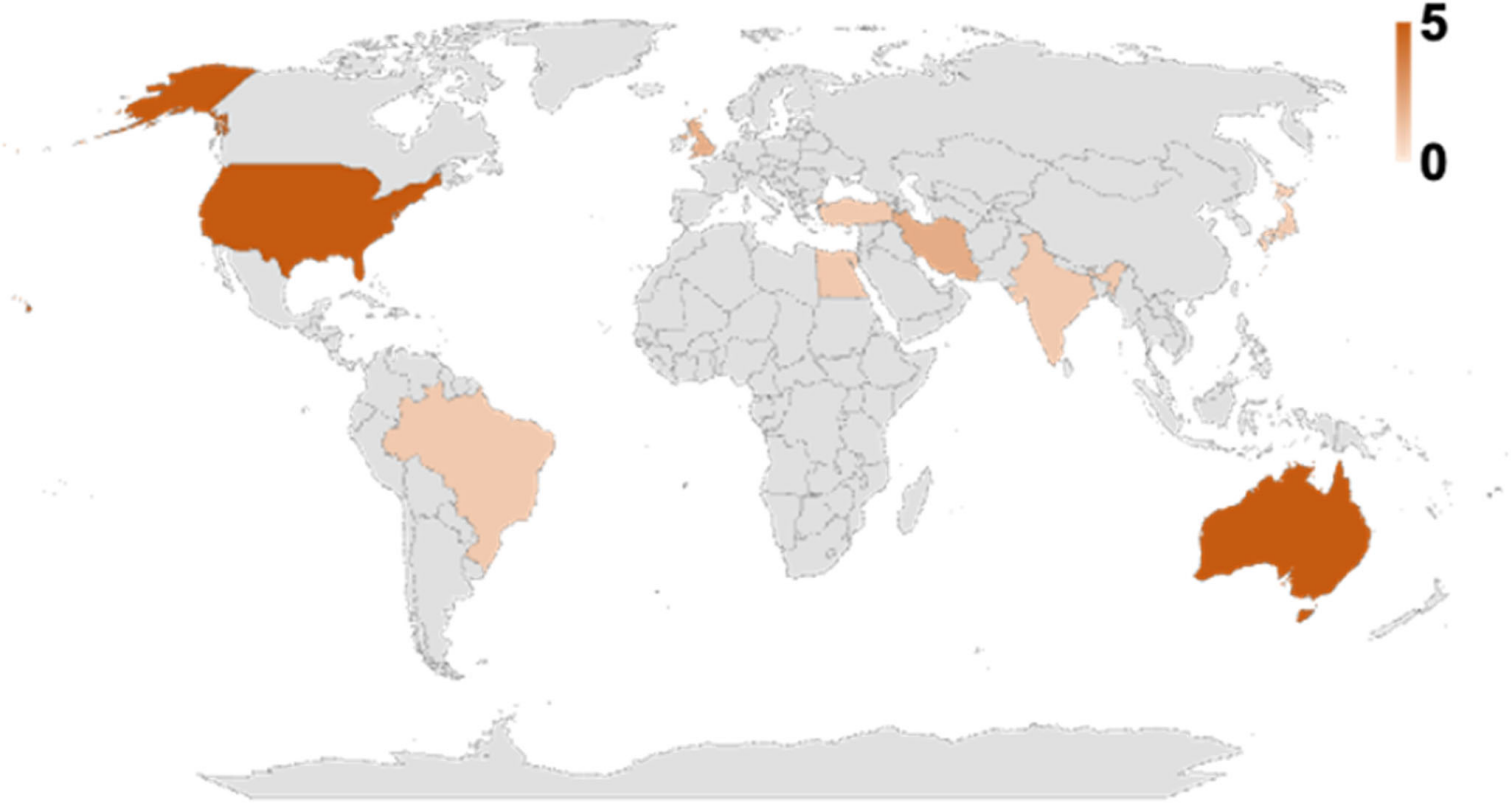
Geographic distribution of the included studies.

The geographical distribution of the 19 included studies is shown in Figure 2. Most of the studies were conducted in the United States (*n* = 5) and Australia (*n* = 5), followed by the United Kingdom (*n* = 2) and Iran (*n* = 2). The other studies were conducted in Brazil, Egypt, Turkey, India, and Japan (*n* = 1 each). This distribution highlights a geographical concentration of the identified literature, with studies originating primarily from the United States and Australia. Further characteristics of the included studies, such as study design and participant details, are provided in Tables 1 (studies with PWS only) and 2 (studies targeting general population samples). The summary rows in these tables report the unweighted mean and median values, with each study treated as an equal unit of analysis. Variability across the studies is described using the reported range (minimum and maximum).

### 3.3 Key Findings

#### 3.3.1 Participant Characteristics and the Proportion of Individuals with a Positive Family History

The included studies were categorised into two groups based on their study populations, namely, those focusing solely on PWS and those focusing on PWS within general population samples. The key characteristics are listed in Tables 1 and 2. Across the included studies, the characteristics of the participants with stuttering were generally consistent with established epidemiological findings. The proportion of male participants was high, with an average of over 70% both in studies focusing solely on PWS and in those targeting general population samples. The onset of stuttering was concentrated in early childhood, with a reported mean age of onset at approximately three to four years. However, most studies (12 out of 19) did not report the mean age of onset. A key finding of this review was the wide variability in the reported proportion of PWS with a family history of stuttering. As shown in Tables 1 and 2, the rates ranged from 20.0% to 90.9% across the studies.

**TABLE 1:** Characteristics of the studies and of the participants when focusing on the persons who stutter (PWS)‐only group.

Authors (year)	Country	Sample size	Mean age (SD) [year]	%Male	Mean age at stuttering onset (SD) [year]	Rate of positive family history of stuttering
Mohammadi and Bakhtiary (2024)	Iran	172	3.7 (0.8)	73.3	3.3 (NR)	69.2
Buck et al. (2002)	UK	61	4.1 (0.9)	68.9	3.0 (0.8)	72.1
Donaher and Richels (2012)	USA	36	10.4 (4.0)	88.9	NR (NR)	61.1
Mohammadi et al. (2016)	Iran	37	9.2 (1.8)	70.3	NR (NR)	90.9
Gürbüz Özgür and Özgür (2019)	Türkiye	64	7.4 (3.8)	70.3	4.9 (2.3)	21.9
Darmody et al. (2022)	Australia	739	NR (NR)	76.5	3.7 (NR)	59.7
Boyce et al. (2022)	Australia	987	NR (NR)	69.6	NR (NR)	49.8
	Mean	299.4	7.0	73.9	3.7	60.7
	Median	64.0	7.4	70.3	3.5	61.1
	Maximum	987	10.4	88.9	4.9	90.9
	Minimum	36	3.7	68.9	3.0	21.9

*Note*: Mean values are calculated as unweighted averages across the included studies; SD, standard deviation; NR, not recorded.

**TABLE 2:** Characteristics of the studies and of the participants when focusing on persons who stutter (PWS) within the general population sample group.

Authors (year)	Country	Sample size	Mean age (SD) [year]	%Male	Mean age at stuttering onset (SD) [year]	Rate of positive family history of stuttering
Choi et al. (2018)	USA	25	3.7 (0.6)	88.0	NR (NR)	24.0
Costa et al. (2022)	Brazil	97	6.6 (2.7)	73.2	NR (NR)	71.1
Nandhini Devi et al. 2018)	India	180	NR (NR)	85.6	4.7 (2.5)	56.1
Sakai et al. (2024)	Japan	128	3.3 (0.2)	56.3	NR (NR)	27.3
Abou Ella et al. (2015)	Egypt	90	NR (NR)	72.2	NR (NR)	31.1
Kefalianos et al. (2017)	Australia	36	NR (NR)	NR	NR (NR)	20.0
Reilly et al. (2009)	Australia	137	NR (NR)	61.3	NR (NR)	51.8
Cavenagh et al. (2015)	UK	42	3.9 (0.8)	64.3	NR (NR)	66.7
Reilly et al. (2013)	Australia	137	NR (NR)	NR	NR (NR)	51.0
Walsh et al. (2021)	USA	21	4.5 (0.6)	81.0	3.1 (0.8)	60.0
Singer et al. (2022)	USA	44	4.1 (0.7)	68.2	2.7 (0.8)	22.7
Viswanath et al. (2004)	USA	56	NR (NR)	78.6	NR (NR)	83.9
	Mean	82.8	4.4	72.9	3.5	47.2
	Median	73.0	4.0	72.7	3.1	51.4
	Maximum	180	6.6	88.0	4.7	83.9
	Minimum	21	3.3	56.3	2.7	20.0

*Note*: Mean values were calculated as unweighted averages across the included studies; SD, standard deviation; NR, not recorded.

#### 3.3.2 Definitions and Assessment Methods in Relation to Family History

The variability in the reported rates of a positive family history appeared to be associated with inconsistencies in the definition and assessment methods. To further explore these potential influencing features, the methodological characteristics of the five studies that reported the highest and lowest proportions of positive family histories were identified and compared, as shown in Tables 3 and 4. This comparison was intended to highlight how specific study characteristics may be related to reported rates in selected cases rather than to demonstrate systematic effects across the entire sample. First, the scope of relatives considered in terms of a family history varied. Studies reporting higher proportions of positive family histories tended to employ a broader definition; for instance, Mohammadi and Bakhtiary (2024) and Costa et al. (2022) included second‐degree relatives (e.g. grandparents, uncles, and aunts), while Buck et al. (2002) and Cavenagh et al. (2015) extended their scope to third‐degree relatives (e.g. cousins). Similarly, Viswanath et al. (2004), who also reported a high proportion, created detailed pedigree charts, although the specific degree of relationship was not explicitly defined. By contrast, studies reporting lower proportions often used a narrower scope. Kefalianos et al. (2017) and Choi et al. (2018) limited their assessment to first‐degree relatives (i.e. parents and siblings) only. In other cases, such as the studies by Gürbüz Özgür and Özgür (2019) and Sakai et al. (2024), the scope of relatives was not clearly reported, rendering direct comparisons difficult. Second, the definition of stuttering among families differed across the studies. Some studies included relatives who had ever stuttered (lifetime incidence, including those who recovered), whereas others included only those who were currently stuttering (prevalence). To further examine this definitional variability, we conducted a descriptive comparison of family history rates according to the definition used for relatives. Specifically, to improve comparability across the studies, we prioritised extracting or calculating data based on the current presence of stuttering (prevalence) in probands when possible. Consequently, the probands were defined based on the current presence of stuttering in 18 of the 19 included studies. Furthermore, the criteria used to classify stuttering among relatives also varied across the studies. Studies using a lifetime definition of stuttering for relatives (*n* = 10) reported a higher mean family history rate (58.71%) than those using a current definition (*n* = 2, 53.3%) or those in which the definition was not clearly reported (*n* = 7, 42.39%). Finally, the data collection methods were not uniform across the studies, which encompassed parent‐reported questionnaires, direct interviews with parents or participants, and telephone reports. The variety of methodologies employed across these studies makes comparison and integration of findings considerably more challenging.

**TABLE 3:** Methodological characteristics of the studies reporting a high proportion of positive family histories.

		Definition of Family History			
Authors (year)	Proportion	Stuttering Status of Proband	Stuttering Status of Relatives	Scope of Relatives	Data Collection Method
Mohammadi et al. (2016)	90.9	Prevalence	Lifetime incidence and prevalence	Up to second‐degree relatives	Parent‐report questionnaire
Viswanath et al. (2004)	83.9	Prevalence	Prevalence	NR (Pedigree chart created)	Telephone report from participant and family
Buck et al. (2002)	72.1	Prevalence	Lifetime incidence and prevalence	Up to third‐degree relatives	Interview with parents
Costa et al. (2022)	71.1	Prevalence	NR	Up to second‐degree relatives	Parent‐report questionnaire
Mohammadi and Bakhtiary (2024)	69.2	Prevalence	Lifetime incidence and prevalence	Up to third‐degree relatives	Interview with parents

*Note*: NR, not recorded.

**TABLE 4:** Methodological characteristics of the studies reporting a low proportion of positive family histories.

		Definition of Family History			
Authors (year)	Proportion	Stuttering Status of Proband	Stuttering Status of Relatives	Scope of Relatives	Data Collection Method
Kefalianos et al. (2017)	20.0	Prevalence	NR	First‐degree relatives	Parent‐report questionnaire
Gürbüz Özgür and Özgür (2019)	21.9	Prevalence	NR	NR	Parent‐report questionnaire
Singer et al. (2022)	22.7	Prevalence	Prevalence	Up to third‐degree relatives	Parent report
Choi et al. (2018)	24.0	Prevalence	Lifetime incidence and prevalence	First‐degree relatives	Interview with parents
Sakai et al. (2024)	27.3	Prevalence	Lifetime incidence and prevalence	NR	Parent‐report questionnaire and interview

*Note*: NR, not recorded.

## 4 Discussion

This scoping review aimed to map the existing literature on family histories in relation to developmental stuttering. The study selection process revealed that there has been an increasing focus on this topic, which is broadly consistent with Yairi and Ambrose's (2013) characterisation of recent work in stuttering epidemiology as reflective of advances occurring in the 21st century. The decision to focus on studies published from 2000 onwards was made to align the review with contemporary research and enhance comparability across studies. The evaluation of the excluded pre‐2000 studies also highlighted differences in study design. While twin studies accounted for 20% of the excluded studies, no twin studies were identified among the included post‐2000 studies. Twin studies can provide important evidence regarding genetic contributions to stuttering; however, their aims and sampling structures differ from those of studies that estimate family history proportions among individuals who stutter. Including such studies in the present synthesis might, therefore, have introduced additional heterogeneity, potentially complicating comparisons of reported family history rates across the studies. However, while the number of studies has clearly increased, this review also highlights considerable methodological variability in the definition of a family history of stuttering and in confirmation methods. Specifically, the findings revealed a notable variability in the reported proportion of individuals with a positive family history, ranging from 20.0% to 90.9%. While participant characteristics were generally consistent with established epidemiological findings, a geographical concentration was observed in the identified literature, with most studies originating from the United States (Choi et al. 2018; Donaher and Richels 2012; Singer et al. 2022; Viswanath et al. 2004; Walsh et al. 2021) and Australia (Boyce et al. 2022; Darmody et al. 2022; Kefalianos et al. 2017; Reilly et al. 2009; Reilly et al. 2013). This finding suggests that research on family history related to developmental stuttering has been conducted primarily in a limited range of countries, highlighting the need for further studies in non‐English‐speaking and non‐Western contexts. However, this pattern should be interpreted with caution, as it may partly reflect our search strategy, which was limited to English‐language publications and selected databases.

### 4.1 Main Findings and Methodological Considerations

A key finding of this review was that the wide variation in the rates of positive family histories appeared to be associated with methodological variability across the studies (Buck et al. 2002; Choi et al. 2018; Reilly et al. 2009). This review identified key sources of this variability, namely, variations in the scope of relatives and in the definitions of the presence of stuttering. To improve comparability across the studies, we prioritised extracting or calculating data based on the current presence of stuttering (prevalence) in probands when possible. Consequently, the probands were defined based on the current presence of stuttering in 18 of the 19 included studies. The criteria used to classify stuttering among relatives varied across the studies. A descriptive comparison across the included studies showed that studies using a lifetime definition for relatives (*n* = 10) reported a higher mean family history rate (58.71%) than those using a current definition (*n* = 2, 53.3%), or those in which the definition was not clearly reported (*n* = 7, 42.39%). Although these comparisons should be interpreted cautiously because of the small number of studies in some categories, the pattern suggests that broader lifetime definitions for relatives may yield higher reported family history rates by capturing relatives who previously stuttered but who no longer do so. This finding further supports the importance of clearly reporting how stuttering is defined among relatives when conducting estimations in relation to family history. The comparability of future studies could be improved by clearly specifying whether the definition used is based on lifetime incidence or prevalence. Additionally, the data collection methods were diverse, and the sources of information were inconsistent across studies. The most commonly used method was the parent‐report questionnaire (i.e., Kefalianos et al. 2017; Mohammadi and Bakhtiary 2024,). Other studies used direct interviews; however, the interviewees varied, with some studies interviewing the parents (i.e., Buck et al. 2002, Özgür and Özgür 2019), while others interviewed the participants themselves (i.e., Özgür and Özgür 2019, Viswanath et al. 2004). This distinction has important implications. While recall bias is a potential risk in any method relying on parental memory (both questionnaires and interviews), the nature of the data and potential biases fundamentally differ when the participant is the direct source of information. This ambiguity in reporting data sources further complicates comparison of the findings. Furthermore, examination of the reporting details suggests a potential systematic bias. For instance, studies reporting higher proportions of a family history of stuttering tended to have fewer instances of ‘Not Reported’ (NR) data for key variables. This feature implies that more rigorous data collection procedures could potentially reveal a higher prevalence of a positive family history. Conversely, it raises the possibility that some studies reporting lower rates may have underestimated the true proportion owing to a less exhaustive investigation. This fundamental inconsistency in methodology and reporting quality remains a challenge when attempting to compare and summarise findings across the literature. Notably, the difference in family history rates observable between Tables 1 and 2 may partly reflect the differences in participant recruitment sources. In this review, approximately 57% of the studies focused exclusively on PWS (Table 1) involving recruited participants from clinical or specialised settings, whereas only 25% of the studies involving general population samples (Table 2) used such recruitment channels. Participants recruited from clinical or specialised settings may differ from those identified through community‐based sampling in terms of stuttering severity or the likelihood that their family history has been recognised or reported. These differences may have contributed to the higher family history rates observed in Table 1 when compared with Table 2.

In addition to these methodological issues, this review also identified a notable gap in the reporting of key clinical data. Most studies (12 of 19) did not report the mean age of onset for their participants. Given that the age of onset is one of the most critical factors for predicting persistence and likelihood of recovery from stuttering, its frequent omission points to an ongoing issue in the consistency of basic data collection, especially in epidemiological studies. This finding highlights the need for future studies to consistently report these core characteristics.

Despite these issues in previous research, the review findings align with that research in certain respects. The confirmation of geographical bias, as hypothesised in the Introduction, is a significant finding. This concentration of research in a few, primarily English‐speaking countries limits the generalisability of the findings and hinders a comprehensive understanding of potential gene‐environment interactions across diverse linguistic and cultural backgrounds (Boyce et al. 2022). However, the consistency of basic epidemiological characteristics, such as the male‐to‐female ratio and the general finding of early childhood onset, with the broader stuttering literature suggests that the studies included in this review captured fundamental aspects in relation to this disorder (Abou Ella et al. 2015; Kefalianos et al. 2017; Nandhini Devi et al. 2018; Reilly et al. 2013; Viswanath et al. 2004).

### 4.2 Limitations and Future Implications

This study had several limitations. The primary objective of this scoping review was to map the extent and nature of the evidence, and not to formally appraise its quality. Therefore, a risk of bias assessment was not performed for the included studies. This implies that the findings should be interpreted as a map of the research landscape and its inherent gaps rather than as a synthesis of the most robust evidence. Screening reliability should also be considered. Although Cohen's kappa indicated moderate agreement, this level of agreement may partly reflect the judgement required when studies mentioned family history but did not clearly report extractable data on the proportion of participants with a positive family history. Looking forward, any call for standardisation must balance ideal rigor with practical feasibility. While investigating third‐degree relatives and including recovered individuals would be ideal for capturing the full genetic picture, the burden on participants and the potential for data inaccuracy must be considered. To enhance the usefulness of family history data, a comprehensive collection of family pedigree data is recommended in clinical practice (Buck et al. 2002). This could involve the development of a tiered system for data collection, in which reporting on first‐degree relatives is considered a minimum requirement, whereas data collection on more distant relatives is recommended based on the study's specific aims and resources. This pragmatic approach would facilitate broader international collaboration and gradually improve the quality and comparability of data in this vital research area.

## 5 Conclusion

This scoping review systematically mapped the existing literature on family histories in relation to developmental stuttering. The review revealed that methodological variation in defining and assessing family histories was the primary factor contributing to the wide variation in reported rates. The findings underscore the importance of developing more consistent research methodologies that enable rigorous comparisons and syntheses in future studies, thereby facilitating greater understanding of stuttering heritability.

## Funding

This work was supported by the Japan Society for the Promotion of Science (JSPS) KAKENHI Grant Number JP25K00007.

## Conflicts of Interest

The authors declare that this study was conducted without any commercial or financial relationships that could be construed as potential conflicts of interest.

## Generative AI Statement

The authors used ChatGPT and Gemini solely for English language editing and proofreading of the manuscript. The authors reviewed and verified all AI‐assisted content and take full responsibility for the final manuscript.

## Data Availability

Derived data supporting the findings of this study are available from the corresponding author, DI, on request.
